# Generating CAR-macrophages to target endothelin B receptor-positive tumors

**DOI:** 10.1007/s00262-025-04219-5

**Published:** 2025-11-12

**Authors:** Cyril Lherminier, Narciso Costa, Amaury Herbet, Marie Hautière, Aloïse Mabondzo, Jérémie Martinet, Didier Boquet

**Affiliations:** 1 CEA, INRAE, Médicaments Et Technologies Pour La Santé (MTS), SPI, Laboratoire d’Etude de L’Unité Neurovasculaire Et Innovation Thérapeutique (LENIT), Université Paris-Saclay, 91191 Gif-Sur-Yvette, France; 2 CEA, INRAE, Médicaments Et Technologies Pour La Santé (MTS), SPI, Laboratoire d’Etudes Et de Recherches en Immunoanalyse (LERI), Université Paris-Saclay, 91191 Gif-Sur-Yvette, France; 3 UNIROUEN, Inserm U1234, FOCIS Center of Excellence, Pan’THER, CHU de Rouen, Department of Immunology and Biotherapy, Normandie Université, 76000 Rouen, France

**Keywords:** Chimeric antigen receptor, CAR-macrophages, Endothelin B receptor, Melanoma, Cancer, Immunotherapy

## Abstract

The endothelin axis is highly involved and overexpressed in many tumors, making it an interesting therapeutic target. However, except for strategies based on small molecule antagonists, this axis is poorly targeted by immunotherapy approaches. Although cell-based therapies, in particular CAR-T cells, have produced impressive results in treating hematological tumors, they remain challenging for solid tumors. CAR-macrophages represent a promising alternative, but only a few therapeutic targets have been evaluated thus far. Having developed antibodies targeting ET1R, our laboratory proposes this axis as a target for CAR-macrophages development. This study investigated the efficacy of CAR-macrophages directed against the endothelin B receptor (ET_B_), expressed by melanoma cells developed from Rendomab B4 (RB4), an antibody targeting ET_B_. Before assembling the CAR against ET_B_, the scFv RB4 fragment, derived from the full-length RB4 antibody, was characterized. It exhibited properties similar to those of the original antibody and displayed exclusive recognition of ET_B_-positive melanoma cell lines. CAR RB4 evaluation showed remarkable antitumor activity against the high ET_B_-expressing WM266 cell line, but no activity on the low ET_B_-expressing A375 cell line. We show the first proof of concept for CAR therapy targeting the endothelin axis and establish CAR RB4 as a promising candidate for the treatment of ET_B_-positive solid tumors.

**Supplementary Information:**

The online version contains supplementary material available at 10.1007/s00262-025-04219-5.

## Background

The endothelin axis is composed of two receptors, the human endothelin type A (ET_A_) and type B (ET_B_) receptor, and three ligands called endothelin (ET-1, ET-2 and ET-3). Several studies have demonstrated the critical role of this signaling pathway in the oncogenesis and progression of various malignant tumors, including glioblastomas, melanomas, ovarian and pancreatic carcinomas [1]. Endothelin receptors have thus become an attractive therapeutic target, as evidenced by over 40 clinical trials in oncology, including recent and ongoing studies (NCT04205227, NCT04158635, NCT05072106). However, the limited efficacy observed in patients may be partly related to the high levels of endothelin in the tumor microenvironment, compromising the action of conventional antagonists [2]. In response to these challenges, we designed and patented the non-antagonist monoclonal antibody Rendomab B4 (RB4), specifically directed against ET_B_, with very promising applications in the treatment of ET_B_^+^ tumors especially melanoma [3].

Cutaneous melanoma represents a growing public health challenge in the world [4]. In 2022, over 100,000 new cases were diagnosed in the European Union-27 (EU-27), resulting in more than 15,000 deaths [5]. According to data from the European Cancer Information System, this type of cancer accounted for 4% of all new cancer diagnoses in 2020, ranking it as the fifth most common cancer in Europe. Melanoma mortality is expected to climb dramatically, rising from 16,736 deaths in 2022 to over 20,900 by 2040. While patients currently have a 93% five-year survival rate, the disease’s tendency for metastasis highlights the critical need for new therapeutic strategies.

The advent of immune checkpoint inhibitor (ICI) immunotherapy has caused a paradigm shift in the management of melanoma, particularly for metastatic cases. Indeed, the use of ICIs elevated five-year survival from 5–19% to over 50% by 2019 [6, 7]. However, these improvements only concern around 40% of patients with metastatic melanoma, highlighting the need to develop new therapeutic approaches [8, 9]. Cellular immunotherapy has emerged as a promising approach for patients ineligible for ICI. The recent clinical trial of lifileucel, a tumor-infiltrating lymphocyte (TIL) therapy, (NCT02360579) validated this approach by demonstrating objective responses over 30% in patients with melanoma refractory to both checkpoint inhibitors and targeted agents, leading to FDA approval of this therapy in 2024 [9, 10]. At the same time, the approval of T-cell therapies genetically modified to express a CAR (Chimeric Antigen Receptor) in hematological cancers in 2017 has generated strong interest for their application in solid tumors [11]. However, clinical trials in melanoma, including the VEGFR2-targeted trial (NCT01218867), have shown no objective response [12]. Hypotheses concerning the failure of these approaches in solid tumors are the low trafficking of T lymphocytes to the tumor, as well as the characteristics of the tumor microenvironment, which limit the efficiency of immune cells [12, 13].

To overcome the trafficking problem associated with the use of CAR-T cells in solid tumors, several publications have explored the potential of other immune cells, in particular CAR-macrophages (CAR-M). The high proportion of macrophages in tumors (TAM) reflects their high migratory capacity and natural ability to infiltrate tumor tissue [14]. Their central function in the communication between innate and adaptive immunity also makes them an ideal candidate for cellular engineering. After having engulfed a target cell, macrophages can present antigens to the rest of the immune system and potentially bypass tumor heterogeneity [15, 16]. All of these factors have contributed to the impressive growth of CAR-M cell research, with 3 clinical trials for the treatment of solid tumors in 2024 (NCT04660929, NCT06254807 and NCT06224738) [17]. Several research teams are also investigating the effectiveness of CAR-M on other target antigens (CEA [18, 19], CD19 [20, 21], EGFRvIII[22], CD147 [23], CD47[24]…), with promising preclinical model results. This research also demonstrated that CAR-M reshape the tumor microenvironment through the secretion of pro-inflammatory cytokines, leading to the reprogramming of M2 macrophages into the M1 phenotype and an increase in CD8+ lymphocyte infiltration [20, 24]. The ET_B_ receptor is highly overexpressed in melanoma, making it a promising immunotherapy target. A recent clinical trial combining the antagonist, ENB-003, with an anti-PD1 antibody is currently underway, highlighting the continued interest in this target (NCT04205227). Given the characteristics of solid tumors, we anticipate that the use of CAR-M targeting ET_B_ could be an effective treatment strategy for melanoma. In this publication, we describe the first generation of CAR-M RB4 against ET_B_, its targeting efficacy and in vitro toxicity on human melanoma cells.

## Methods

### ScFv expression and purification

The variable heavy (VH) and variable light (VL) chains of the RB4 antibody were fused with a linker peptide ((GGGGS)_4_). An 8xHIS tag was added to the C-terminus of the scFv for purification purposes. The scFv RB4 sequence was subcloned into a eukaryotic expression vector (pTT5) and expressed in ExpiCHO-S cells (Thermo Fisher Scientific, A29127). The scFv was purified from the supernatant using a HisTrap high-performance column (GE Healthcare, 17-5248-02). Purification was confirmed by SDS-PAGE.

### CHO cells

Three established Chinese hamster ovary (CHO) cell lines were used: CHO-WT, CHO-ET_A_ and CHO-ET_B_. The CHO-ET_A_ and CHO-ET_B_ cell lines were generated by transfection of the CHO-K1 cell line (ECACC) with the pcDNA3.1 vector (Invitrogen) encoding human ET_A_ or ET_B_, using FuGENE HD reagent (Roche Diagnostics, E2311), followed by selection in the presence of 1 mg mL⁻^1^ G418 (Gibco, 10,131,027). Cells were cultured in HAM-F12 medium (Gibco, 21,765) supplemented with 10% fetal bovin serum (Gibco, 17,479,633), 1 mM pyruvate (Gibco, 11,360), 1% non-essential amino acids (Gibco, 11,140), 2 mM glutamine (Sigma, G7513), 100 U mL^−1^ penicillin and 100 µg mL^−1^ streptomycin (Sigma, P0781). The stable expression of ET_A_ and ET_B_ receptors was maintained with 0.5 mg mL^−1^ of geneticin. Cells were incubated at 37 °C with 5% humidified atmosphere.

### Tumor cells

Melanoma cell line UACC257, purchased at DCTD Tumor Repository (Frederick, Maryland, USA), was cultured in RPMI-1640 (Sigma, R0883). Melanoma cell lines A375 and WM266, (ECACC, England), were cultured in DMEM (Gibco, 31,331,093). All media were supplemented with 10% fetal bovin serum (Gibco, 17,479,633), 1 mM pyruvate (Gibco, 11,360), 1% non-essential amino acids (Gibco, 11,140), 2 mM glutamine (Sigma, G7513), 100 U mL^−1^ penicillin and 100 µg mL^−1^ streptomycin (Sigma, P0781). Cells were maintained at 37 °C in a humidified atmosphere of 5% CO2.

### Binding experiments by flow cytometry

To determine the apparent dissociation constant (Kd) and assess specificity, binding experiments were performed using 1.5 × 10^5^ living cells per sample, resuspended in saturation buffer (PBS-1x, 5% normal goat serum (NGS) and 0.1% bovine serum albumin (BSA)) at concentrations ranging from 0.05 to 1,500 nM, according to a previously described protocol [3, 25]. For binding curves, each range point was incubated with cells at 4 °C overnight. For revelation, 6x-His Tag Monoclonal Antibody conjugated with Alexa Fluor 488 (Invitrogen, MA1135A488) were diluted at 1/200 in saturation buffer and incubated during 3 h at 4 °C. Three washes with PBS 1X (pH = 7.4) were performed between each step. The same procedure was used to check the specificity on CHO-ET_B_ cells after incubation but with the addition of a final concentration of 100 nM of each endothelin to induce internalization of the ET_B_ receptor and reduce its number on the cell membrane. Experiments were read with the FACSCalibur flow cytometer (BD Biosciences, NJ, USA). Significance was determined by an unpaired t test performed on points from the saturation phase. We normalized MFI data from 0 to 100%, where 0% corresponds to cell autofluorescence and 100% to the saturated MFI signal on CHO-ET_B_ cells. Curve fitting was performed using the site-specific binding model. Data are presented as mean ± SEM.

### Generation of CAR RB4 cell line

CAR RB4 expression in THP-1 cell was introduced by transduction with our CAR RB4 lentivirus (CD8 signal peptide, scFv RB4 targeting ET_B_ receptor, CD8 transmembrane domain, CD28 co-stimulation domain and CD3z stimulation domain), produced by VectorBuilder (Chicago, IL, USA). The structural map and sequences of the domains composing CAR RB4 are available in Supplementary Fig. 1A, B. 2.5 × 10^5^ THP-1 cells were cultured in a 24-well plate and transduced in a final volume of 500 µL of RPMI-1640 complete medium containing 4 µg/mL polybrene (VectorBuilder) at a MOI of 5. After incubation for 24 h at 37 °C under 5% CO_2_, 500 µL of fresh RPMI medium was added. 48 h after transduction, cells were washed three times in PBS by centrifugation (200 g, 5 min), and then, culture was performed using normal conditions. Transduction efficiency was assessed 5 days post-transduction by flow cytometry, using the anti-G4S antibody Alexa Fluor 488 (T-Cell Signaling, G4S Linker Rabbit mAb, 50,515). Finally, CAR RB4-positive cells were isolated by cell sorting on a FACSMelody™ Cell Sorter (BD Biosciences, USA) using the anti-G4S antibody.

### Analysis of ETB receptor expression on tumor cell lines

The expression of the ET_B_ receptor was analyzed by RT-qPCR and confirmed by flow cytometry. For RT-qPCR experiments, total RNA was extracted from cell pellets using the RNeasy Mini Kit (Qiagen-74104), according to the manufacturer’s instructions. The concentration and quality of extracted RNA were then verified by spectrophotometry (NanoDrop, Thermo Fisher Scientific). Reverse transcription was performed on 1 µg of RNA using the High-Capacity cDNA Reverse Transcription kit (Applied Biosysteme-4368813) according to the manufacturer’s recommendations. qPCR was performed on the CFX96 PCR detection system (Bio-rad, CA, USA) using iTaq Universal SYBR Green Supermix (Bio-rad, 1,725,121) and primers available in Supplementary Fig. 2A. Each reaction was performed on 12 µL with a final primer concentration of 10 µM. Prior to qPCR realization, a housekeeping gene test was performed on a gene set (CASC3, RSP2, PPIA, TBP, EIF4A, YWHAZ, SDHA, GAPDH) to select TBP and EIF4A for normalization of gene expression (Supplementary Fig. 2B and C). For flow cytometry, RB4 antibody (RB4 mAb) was used at a final concentration of 30 nM, then revealed for 3 h with anti-mouse goat antibody Alexa Fluor™ 488 (Invitrogen™, A10684) diluted at 1/200. Experiments were read with the Attune NxT flow cytometer (Thermo Fisher Scientific, USA) and analyzed with floreada.io.

### Tumor cell lines targeting analysis

The targeting of tumor cell lines A375, UACC257 and WM266 was analyzed by fluorescence microscopy using the ZOE microscope (ZOE Fluorescent Cell Imager, Bio-rad, 1,450,031). Tumor cells were labeled in green with CellTracker CFSE (Invitrogen, C34554) at a concentration of 5 µM, according to the manufacturer’s recommendations and then seeded in a 12-well plate at 7.5 × 10^4^ cells per well. After 24 h of incubation (37 °C, 5% CO2), the culture medium was replaced, and THP-1 or CAR RB4-expressing THP-1 monocytes (CAR RB4) were added at an effector/target (E/T) ratios of 1:1 and 5:1. After four hours of co-culture, the different conditions were observed under microscope and photographed. Images were captured randomly in each well and analyzed manually to quantify the number of monocytes interacting with tumor cells (green color). The targeting coefficient (number of targeting monocytes/number of tumor cells) was then calculated and normalized to the control condition (THP-1). Data analysis was carried out using GraphPad v10.5.0, and statistical tests were performed using one-way ANOVA, with data tested for normality.

### Polarization of the THP-1 cell line into M0 macrophages

Polarization of the THP-1 cell line into M0 macrophages was performed in the presence of 80 ng/mL PMA (Sigma-P8139) for 24 h, at a concentration of 5 × 10^5^ cells/mL, on TC-treated Cell Culture Dish (Falcon™ 353,003). Only cells that adhered to the support were used in further experiments. The primers used for RT-qPCR are available in Supplementary Fig. 3A. Differentiation using the markers CD11b and CSF1R was validated (as previously described [26]), and PPIA and SDHA housekeeping genes were selected from a set of genes (TFRC, SHGA, PPIA, RPL13A, RPLP0, CASC3, RPS2, GAPDH) (Supplementary Fig. 3B and C).

### Analysis of phagocytosis and tumor growth inhibition in a 2D model

Phagocytosis was studied by flow cytometry using an in vitro co-culture model. To identify tumor cells, A375, UACC257 and WM266 cell lines were modified by lentiviral transduction to express mCherry fluorescent protein (mCherry) and a puromycin resistance gene. Transduction was performed at an MOI of 5, and fluorescent cells were then selected by puromycin (2 µg/mL) for 4 days. After selection, ET_B_ receptor expression was assessed by RT-qPCR as previously described, using the same housekeeping genes and primers. Tumor cells A375 (ET_B_^low^) and WM266 (ET_B_^high^) expressing fluorescent mCherry proteins were then used as targets. THP-1 or CAR RB4 macrophages, generated from the THP-1 cell line after PMA polarization, were used as effectors and pre-labeled with 5 µM CellTrace Violet (Thermo Fisher, C34557), according to the manufacturer’s recommendations. Co-cultures were performed at an E/T ratio of 1:1 and 5:1 for 72 h. Cells were then detached with Versene, washed (PBS 1X), fixed and analyzed by flow cytometry (Attune NxT, Thermo Fisher Scientific). All steps were carried out using identical volumes to ensure absolute counting. Macrophages that had phagocytosed tumor cells were identified as the Violet⁺/mCherry⁺ double-positive population. Tumor growth was analyzed by identifying tumor cells as the Violet-/mCherry⁺ population. All conditions were performed with *n* = 6. Experiments were read with the Attune NxT flow cytometer (Thermo Fisher Scientific, USA). Data were analyzed using Floreada.io, excluding debris and by FSC/SSC gating. The different conditions were normalized to the condition containing unmodified THP-1 macrophages.

### Analysis of targeting and cytotoxicity in a 3D model

Spheroids were generated from melanoma cell line WM266 (ET_B_^high^) by seeding 5.10^3^ tumor cells per well in low-adhesion 96-well plates (Corning, CLS7007) in 200 µL of complete medium. Cells were incubated 48 h at 37 °C, 5% CO_2_ to allow the formation of 130.000 µm^2^ spheroids. Tumoroids were then transferred to 24-well low-adhesion (Sarstedt, 83.3922.500) plates and co-cultured with THP-1 or CAR RB4 macrophages at an E/T ratio of 5:1. Effector cells were labeled in green with CellTrace CFSE to allow visualization of macrophages targeting of WM266 cells after 1 h of co-culture by fluorescence microscopy (ZOE Fluorescent Cell Imager, 1,450,031). After 7 days of co-culture, the cells making up the tumoroid/macrophages mixture were dissociated by aspiration and flushing and then washed in PBS. The cells were incubated with 7-AAD (Biolegend, 420,403) for 10 min, protected from light, to study the percentage of viable cells in the WM266 cell population. The number of cells composing the tumoroid was also studied after exclusion of the macrophage population by SSC/Celltrace CFSE gating. Experiments were read with the Attune NxT flow cytometer (Thermo Fisher Scientific, USA) and analyzed with floreada.io.

### Statistics

Statistical analyses were performed using GraphPad Prism v10.5.0. For binding experiments based on flow cytometry, a normality test was performed on all points of the saturation phase, followed by a nonparametric *t* test to compare the different conditions. For all other analyses, depending on sample size, a *t* test or two-way ANOVA was performed after evaluation of normality and homogeneity of variances to ensure appropriate model selection.

## Results

### scFv RB4 retains the same affinity and specificity as the full antibody

#### An antibody fragment specific to the ETB receptor

scFv RB4, derived from the fusion of the VH and VL variable regions of the RB4 murine antibody, was produced in ExpiCHO cells and purified on a histidine column. The size and purity of the fragment produced were checked first by SDS-PAGE (Supplementary Fig. 4A). On the line containing the scFv RB4 after purification, the presence of a single band at 25 kDa validated the purity of the fragment and the purification process. To validate the reproducibility of production, three productions were carried out and compared through the analysis of thermal denaturation profiles (Supplementary Fig. 4B). All three fragments showed identical denaturation profiles, with a single inflection temperature of 60.7 °C.

**Fig. 1:**
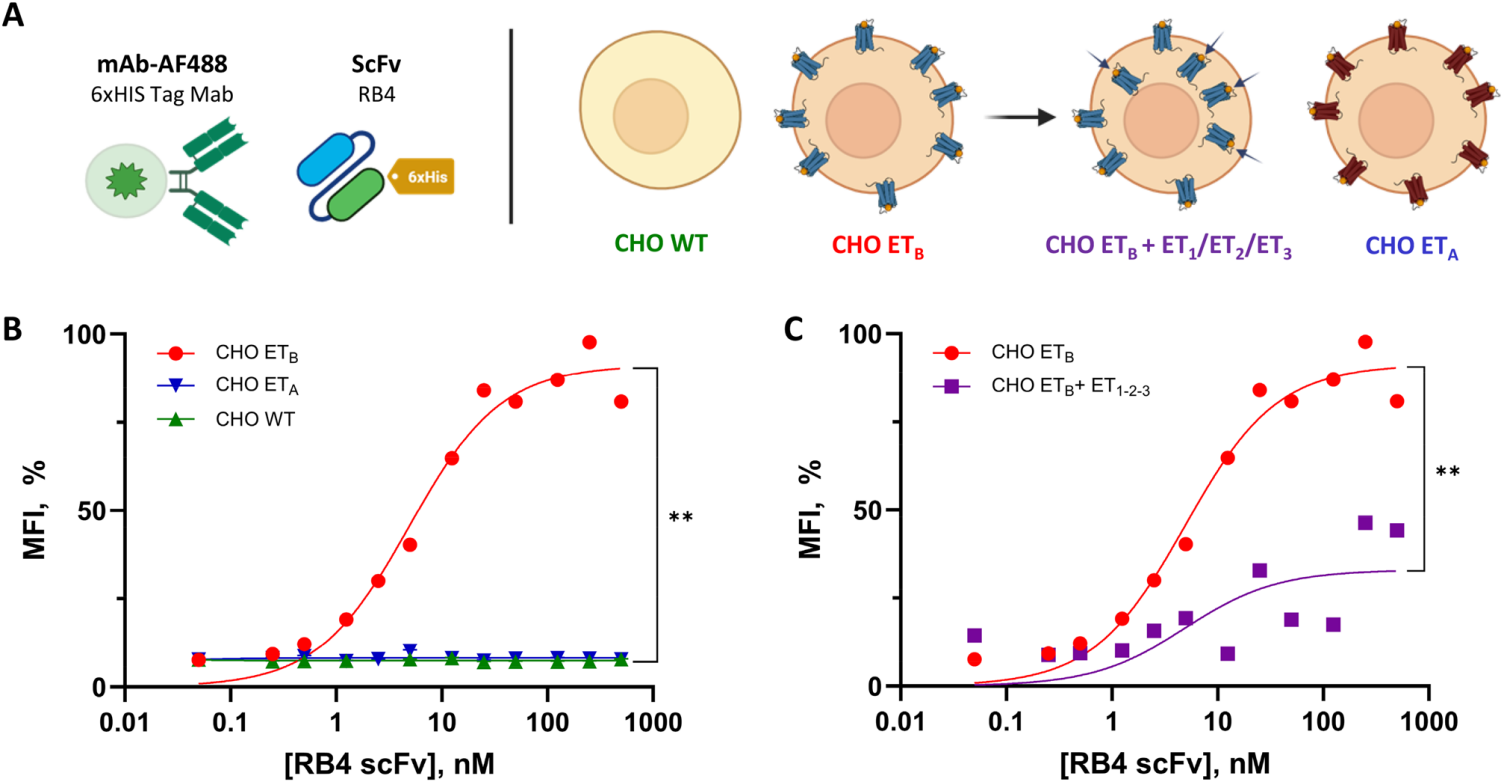
Evaluation of scFv RB4 affinity and specificity in ET_B_ receptor overexpressing cells. **A** Schematic representations of the experimental approach. **B** Schematic representation of scFv binding experiments on CHO cell lines: CHO-WT (green), CHO-ET_A_ (blue) and CHO-ET_B_ (red). Binding curves were generated by plotting normalized MFI (%MFI) versus scFv concentration. Differences were observed between CHO-ET_B_ and each condition (*p*.value = 0.0022). **C** ScFv binding curves on CHO-ET_B_ without or with high concentrations of ET, ET-1, ET-2 and ET-3 at 100 nM each. scFv RB4 binding is partially abolished at high ET concentrations. A difference was observed between the two conditions (*p*.value = 0.0022)

Figure 1A illustrates the model used to validate the specificity and affinity of the scFv RB4 fragment for ET_B_. Briefly, CHO cells overexpressing endothelin A and B receptors (CHO-ET_A_, CHO-ET_B_) versus CHO wild type (CHO-WT) were used to demonstrate the binding specificity of the scFv RB4 against ET_B_ as previously described.[3, 25]. Figure 1B illustrates the binding curves of the RB4 scFv on different cell models. As expected, the increase in fragment concentration did not induce a modification in fluorescence on CHO-WT and CHO-ET_A_ cells, in contrast to the red profile observed on CHO-ET_B_. The increase in scFv RB4 concentration leads to a rise in MFI from the third measurement (0.25 nM) to the eighth measurement (12.5 nM). Above 25 nM, the MFI seems to reach a plateau, and increasing the concentration of the RB4 fragment no longer leads to an increase in fluorescence. Binding experiments show that the fragment binds only to CHO-ET_B_ cells with an apparent dissociation constant (Kd)of 4.9 nM. The comparison of “saturation” phases between the different curves shows a significant difference between the CHO-ET_B_, CHO-ET_A_ and CHO-WT (*p*.value = 0.0022, **) (Supplementary Fig. 5A).

To demonstrate the specificity of the fragment for ET_B_, we induced the internalization of the receptor in the presence of a large excess of endothelin. The two binding curves are presented (Fig. 1C), one (dotted red curve) obtained on CHO-ET_B_ cells with 100 nM of each endothelin (CHO-ET_B_+ET_1-2-3_) resulted in a smaller increase in fluorescence than in CHO-ET_B_ cells alone (red curve). The addition of endothelin prior to the binding experiments is responsible for an 64% reduction in maximum MFI underlying the specificity of the scFv RB4 for the human ET_B_ receptor (91.18 for CHO-ET_B_ vs. 32.99 for CHO-ET_B_+ET_1-2-3_) (p.value = 0.0022, **) (Supplementary Fig. 5A and B).

#### scFv RB4 can recognize melanoma cells overexpressing the ETB receptor

**Fig. 2:**
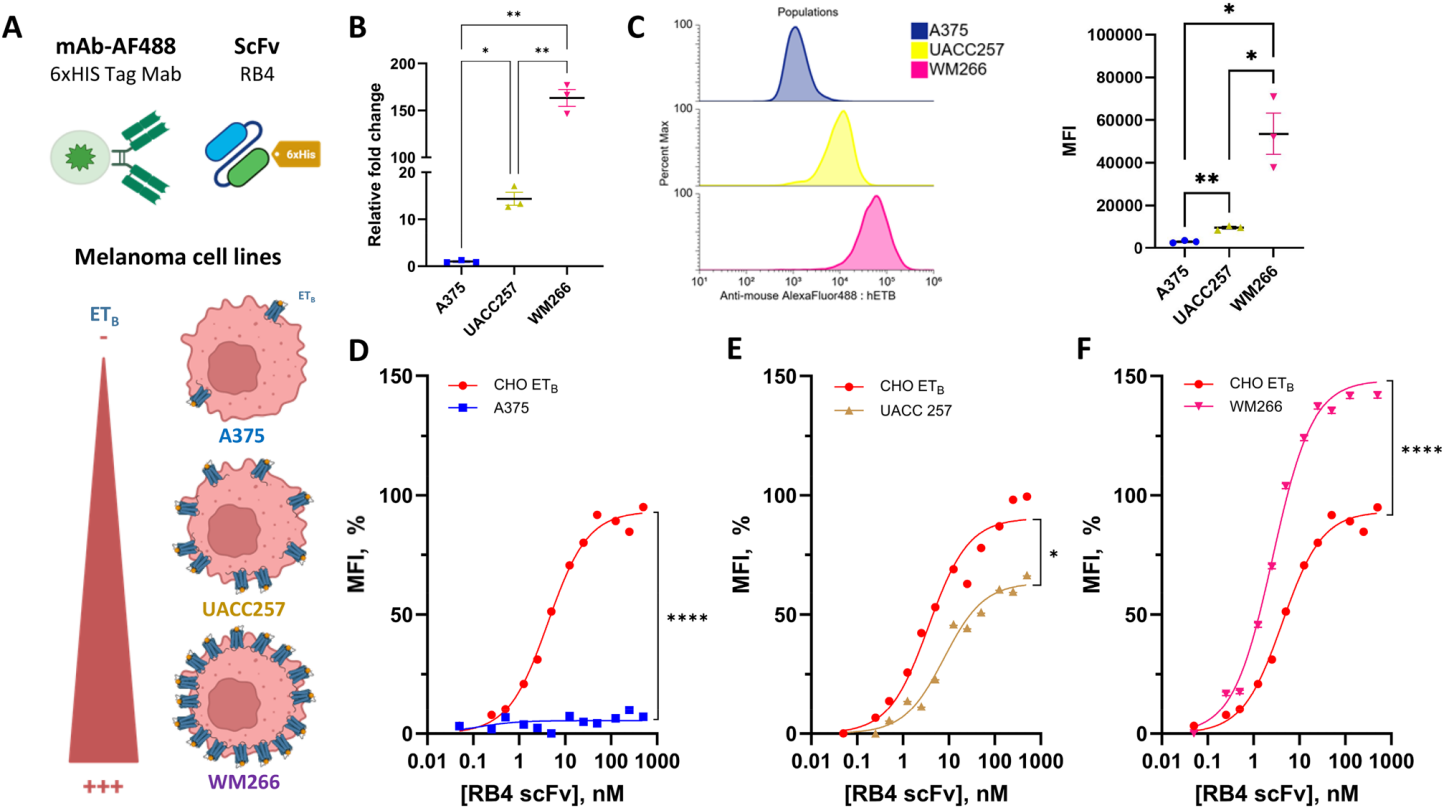
Evaluation of scFv RB4 affinity on human melanoma tumor lines **A** Schematic representations of the experimental approach and cellular model. **B** Endothelin B receptor (ET_B_) expression in A375, UACC257 and WM266 melanoma cell lines analyzed by RT-qPCR. Differences were observed between each cell lines (A375 vs. UACC257, *p*.value = 0.0225; A375 vs. WM266, *p*.value = 0.0065; UACC257 vs. WM266, *p*.value = 0.0079). **C** Flow cytometric analysis of ET_B_ expression in A375 (blue, MFI = 3,001 ± 566), UACC257 (yellow, MFI = 9,484 ± 1,064) and WM266 (purple, MFI = 53,539 ± 16,660) cell lines. Analysis was performed using RB4 mAb. Differences were observed between each cell lines (A375 vs. UACC257, *p*.value = 0.0024; A375 vs. WM266, *p*.value = 0.0342; UACC257 vs. WM266, *p*.value = 0.0440). **D**–**F** Schematic representation of scFv binding experiments on CHO-ET_B_ (red) and melanoma cell lines: **D** WM266 (purple), **E** UACC 257 (yellow) and **F** A375 (blue). Significant differences were observed CHO-ET_B_ vs. WM266 (*p* < 0.0001), CHO-ET_B_ versus UACC (*p* = 0.0132) and CHO-ET_B_ versus A375 (*p* < 0.0001)

Melanomas were recognized as cancers involving deregulation of the endothelin axis and the overexpression of the ET_B_. As illustrated in Fig. 2A, three human melanoma cell lines were selected: A375, UACC257 and WM266, which have low, intermediate and high ET_B_ levels, respectively, as confirmed by RT-qPCR (Fig. 2B). Gene expression levels were first normalized to the housekeeping genes TBP and EIF4A. Next, expression values were compared against the A375 cell line, which was defined as the baseline (relative fold change = 1). Using this method, ET_B_ expression in UACC257 and WM266 cells showed relative fold changes of 14.36 and 163.30, respectively, indicating higher expression compared to A375. These data confirm the expression of ET_B_ in these cellular models, which will be used in subsequent experiments. The expression of ET_B_ in melanoma cell lines was also complemented by FACS analysis using the RB4 antibody (Fig. 2C). A375 showed the lowest fluorescence (MFI = 3,001 ± 566), followed by UACC257 (MFI = 9,484 ± 1,064) and WM266 (MFI = 53,539 ± 16,660). These results are in agreement with RT-PCR gene expression (Fig. 2B). Therefore, we classified the three human melanoma cell lines as follows: A375 (ET_B_^low^), UACC257 (ET_B_^medium^) and WM266 (ET_B_^high^).

Binding experiments were carried out on melanoma cell lines and compared with CHO-ET_B_. Low fluorescence level was observed on A375 (Fig. 2D) as previously observed with the RB4 mAb (Supplementary Fig. 6B). Analysis of the binding curves, between the three cell lines, highlights the differences in expression between the cells. Low binding was observed on A375, while the B_(max) values (Supplementary Fig. 5D) were found to be 63.40% for UACC257 (Fig. 2E) and 148.30% for WM266 (Fig. 2F). Furthermore, analysis of fluorescence averages over the saturation phases demonstrates significant differences between the three binding profiles (*p*.value < 0.0001 for « A375 vs. ET_B_» and « WM266 vs. ET_B_», *p*.value = 0.0132 for « UACC257 vs. ET_B_»).

The apparent affinities obtained with the different formats are summarized in Supplementary Fig. 6C. The reduced apparent affinity of the scFv fragment (Kd 4.9 nM) compared with the mAb RB4 (Kd 0.15 nM) may be partly attributable to the lack of bivalence of the scFv RB4. Other structural differences, such as the presence of the G4S linker, could also explain the change in binding properties.

In conclusion, scFv RB4 is capable of recognizing melanoma cells expressing the endothelin B receptor (ET_B_) from a certain level of expression, such as that observed by UACC257.

### CAR RB4 specifically targets ETB^+^ melanoma cells

After validating the functionality of the scFv RB4 fragment (affinity, specificity and recognition of ET_B_^+^ melanoma cell lines), it was inserted into a second-generation CAR structure with a CD8 hinge and CD28 and CD3z as stimulation domains (Supplementary Fig. 1).

**Fig. 3:**
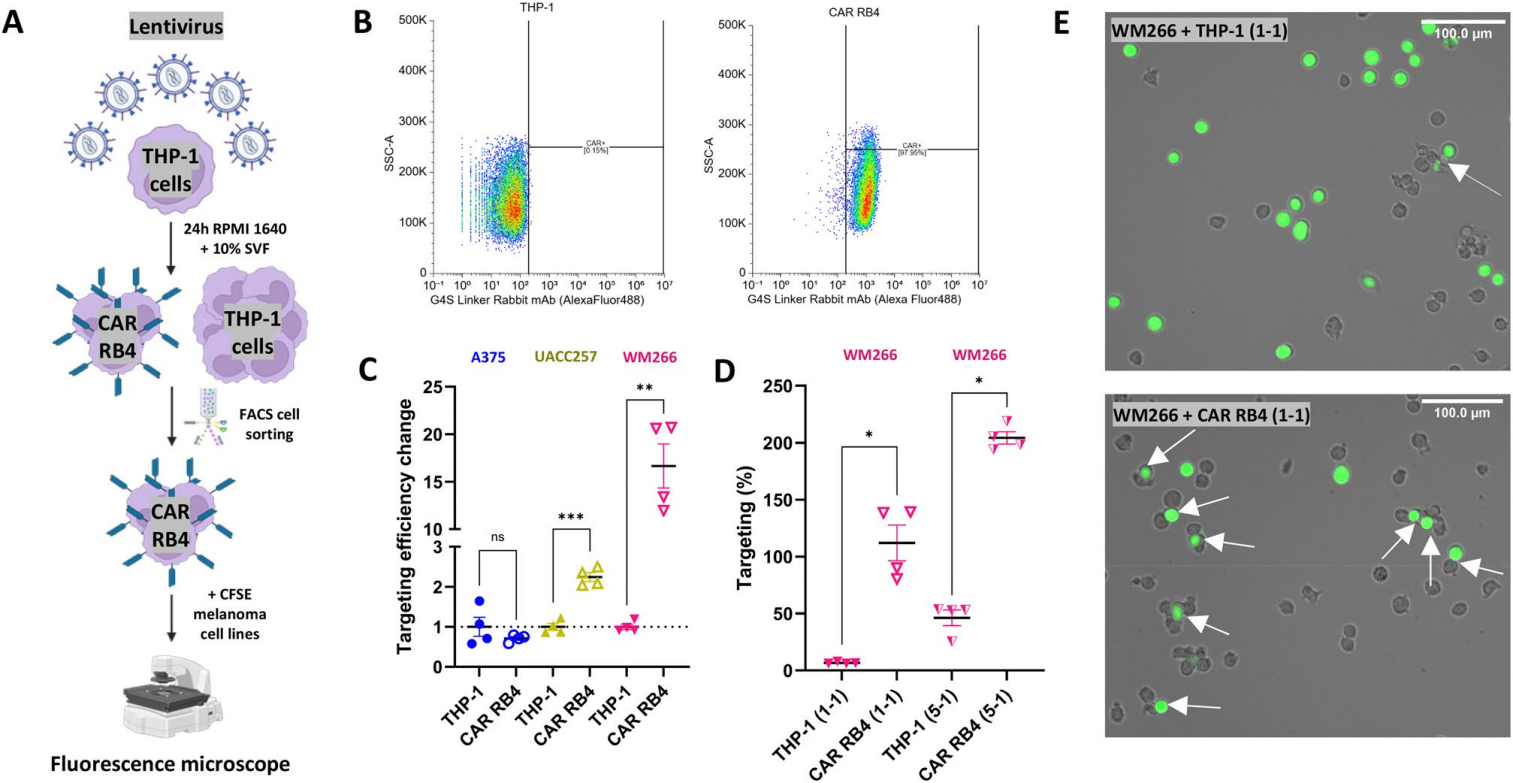
Evaluation of THP-1 CAR targeting in human melanoma cell lines **A** Schematic representations of the experimental approach to developing the CAR RB4 cell line. **B** Flow cytometry analysis of CAR RB4 expression after cell sorting of CAR RB4 THP-1 cell line. Data were plotted as SSC-A versus Alexa Fluor 488 fluorescence profiles using the G4S anti-linker antibody. **C** Efficacy of THP-1 and CAR RB4 monocytes targeting against A375 (blue), UACC257 (yellow) and WM266 (purple) cell lines at an E/T ratio of 1:1. Data were normalized with THP-1 condition for each cell line. Differences were observed between THP-1 and CAR RB4 for UACC257 (p.value = 0.0001) and WM266 (*p*.value = 0.0066). **D** Targeting rate of the WM266 line by CAR RB4 for E/T ratios of 1:1 and 5:1. Differences were observed for each condition (*p*.value = 0.0175). **E** Microscopic observation and targeting analysis of WM266 tumor cells (CFSE + green cell) by THP-1 or CAR RB4 monocytes at an E/T ratio of 1:1. Target areas are indicated by white arrows

Figure 3A illustrates the process adopted to generate a population of CAR RB4 THP-1 monocytes using lentiviral transduction. The anti-linker antibody was used to sort CAR RB4 by fluorescence-activated cell sorting (FACS) after validating the absence of binding on the THP-1 cell line (Supplementary Fig. 7A). Figure 3B illustrates the binding of the anti-linker antibody to either the THP-1 monocyte (left side) or the purified CAR RB4 (right side). Analysis of the cytometry data shows that the CAR RB4+ population is enriched to 97.95%, thus validating the modified cell line. The impact of the lentivirus on the expression of the CD14, CD11b and CSF1R biomarkers was also quantified at transcript and protein levels (Supplementary Fig. 8). A small variation in mRNA expression was detected for all biomarkers, but no significant effect was detected at the protein level. It was therefore concluded that lentivirus had no significant influence on the activation status of the CAR RB4 cell line.

Each melanoma cell line is recognized differently by unmodified THP-1 cells due to their allogeneic and tumor origin. To specifically evaluate the contribution of CAR RB4 independently of background recognition by THP-1 cells, the CAR cell response was quantified and normalized relative to the THP-1 cell line background signal. We initiated co-culture experiments to evaluate the efficacy of CAR RB4 targeting, using an E/T ratio of 1:1 (Fig. 3C). CAR RB4 significantly targeted UACC257 (yellow data) by a factor of 2 and targeting increased dramatically for WM266 (purple data) by a factor of 17. Conversely, there was no increase in targeting for A375, which is why it was used as a negative control in the subsequent experiments. These results confirm the link between ET_B_ receptor expression and CAR RB4 targeting efficiency. To evaluate the impact of an increased E/T ratio on CAR RB4 targeting efficiency, the 1:1 ratio was compared with the 5:1 ratio on WM266 cells (Fig. 3D). All conditions were normalized to the THP-1 1:1 ratio condition. With a 1:1 ratio, CAR RB4 achieves 112% targeting versus 204% for the 5–1 ratio. This E/T ratio is also associated with increased non-specific targeting of THP-1 cells (6.72% for 1:1 ratio and 46.29% for the 5:1 ratio). A similar analysis was carried out using an E/T ratio of 5:1 on each melanoma cell line to investigate the impact of a higher E /T ratio on targeting efficiency (Supplementary Fig. 9). Contrary to expectations, increasing the E/T ratio did not lead to any further improvement in tumor cell targeting, as targeting of all cells was already achieved with a 1:1 ratio and additionally non-specific targeting increased with the 5:1 ratio. Representative images of THP-1 and CAR RB4 targeting WM266 cells at a 1:1 ratio are shown (Fig. 3E). Targeting sites are indicated by white arrows. CAR RB4 exhibits a greater number of targeting events than unmodified THP-1 monocytes.

### CAR RB4 induces an antitumor response against ETB^+^ cells

#### *CAR RB4 increases phagocytosis rates and inhibits tumor growth in ETB*^+^*cells in 2D model.*

**Fig. 4:**
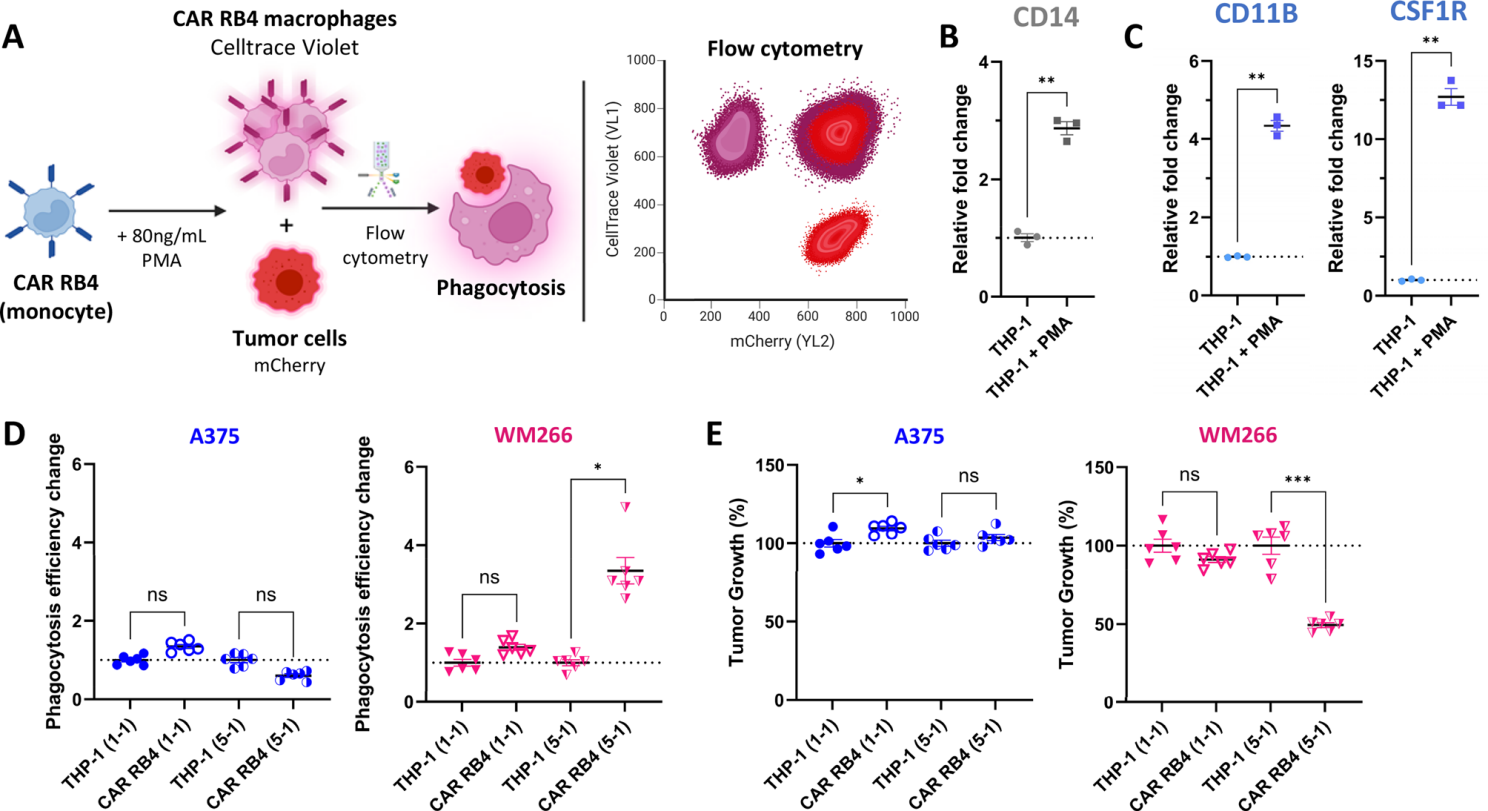
Evaluation of cytotoxicity and phagocytosis in human melanoma cell lines **A** Schematic representations of the experimental approach to analyze phagocytosis and tumor growth in 2D model. **B**-**C** Analysis of THP-1 polarization using 80 ng/mL PMA for CD14, CSF1R and CD11B biomarkers. Differences were observed for each condition (CD14, *p*.value = 0.0031; CD11b, *p* value = 0.0059; CSF1R, *p* value = 0.0067). **D** Phagocytosis rate of A375 (blue) and WM266 (yellow) cells by THP-1 macrophages expressing or not CAR RB4. Phagocytosis was defined as CellTrace Violet + /mCherry + events. No difference was observed for the A375 cell line. The only difference was observed in the WM266 at an E/T ratio of 5:1 (*p*.value = 0.0338). **E** Relative tumor growth of A375 and WM266 cells co-cultured with THP-1 or CAR-THP-1 macrophages. No difference was observed for the A375 cell line. Significant inhibition was observed only for the WM266 cell line at the 5:1 ratio (*p*.value = 0.0004)

To assess the cytotoxicity generated by the CAR RB4, a co-culture model comprising mCherry fluorescent A375 and WM266 tumor cells and CellTrace Violet-stained CAR RB4 macrophages was employed (Fig. 4A). Firstly, to induce macrophages, PMA polarization on THP-1 was performed and validated by RT-qPCR (Fig. 4B and C). CD14, CSF1R and CD11b biomarkers were assessed on cells before and after polarization. Overall, there was a significant increase in all biomarkers, particularly for CSF1R, showed by a relative change in expression greater than tenfold, validating the differentiation of monocytes into macrophages. Next experiments were carried out using differentiated macrophages and fluorescent tumor cells and analyzed 72 h after co-culture. We also examined the presence of ET_B_ expression by RT-qPCR after the introduction of mCherry by a lentivirus in fluorescent tumor cells. EDNRB gene expression levels in A375 mCherry cells are 450-fold lower than in WM266 mCherry cells (Supplementary Fig. 10), validating the expression profile in mCherry cells in a manner consistent with unmodified cells. Figure 4D and E shows the results for changes in the rate of phagocytosis and tumor growth, respectively. Similar to the results shown in Fig. 2, phagocytosis and tumor growth analyses were normalized to a condition containing the same ratio of unmodified THP-1 cells in order to study only the CAR-mediated effect. For the modification of the phagocytosis rate (Fig. 4D), no significant modification is observed for A375 at either the 1–1 or 5–1 ratio, in contrast to WM266. A significant 3.35-fold increase in phagocytosis rate was observed for WM266 at a 5–1 ratio compared with the THP-1 condition. Thus, it can be concluded that the increase in phagocytosis is present only on ET_B_^+^ cells at a 5–1 ratio. Similar results were obtained for tumor growth (Fig. 4E). No significant difference in tumor growth is found for A375 on the two ratios, while an inhibition of around 50.6% of WM266 growth is present in the CAR RB4 condition compared with THP-1 always at 5–1 ratio. Unlike targeting efficiency, which was already maximal at an E/T ratio of 1:1, a 5:1 ratio was necessary to induce a significant antitumor cytotoxic effect.

#### *CAR RB4 inhibits tumor growth of ETB*^+^*cells in 3D model.*

**Fig. 5:**
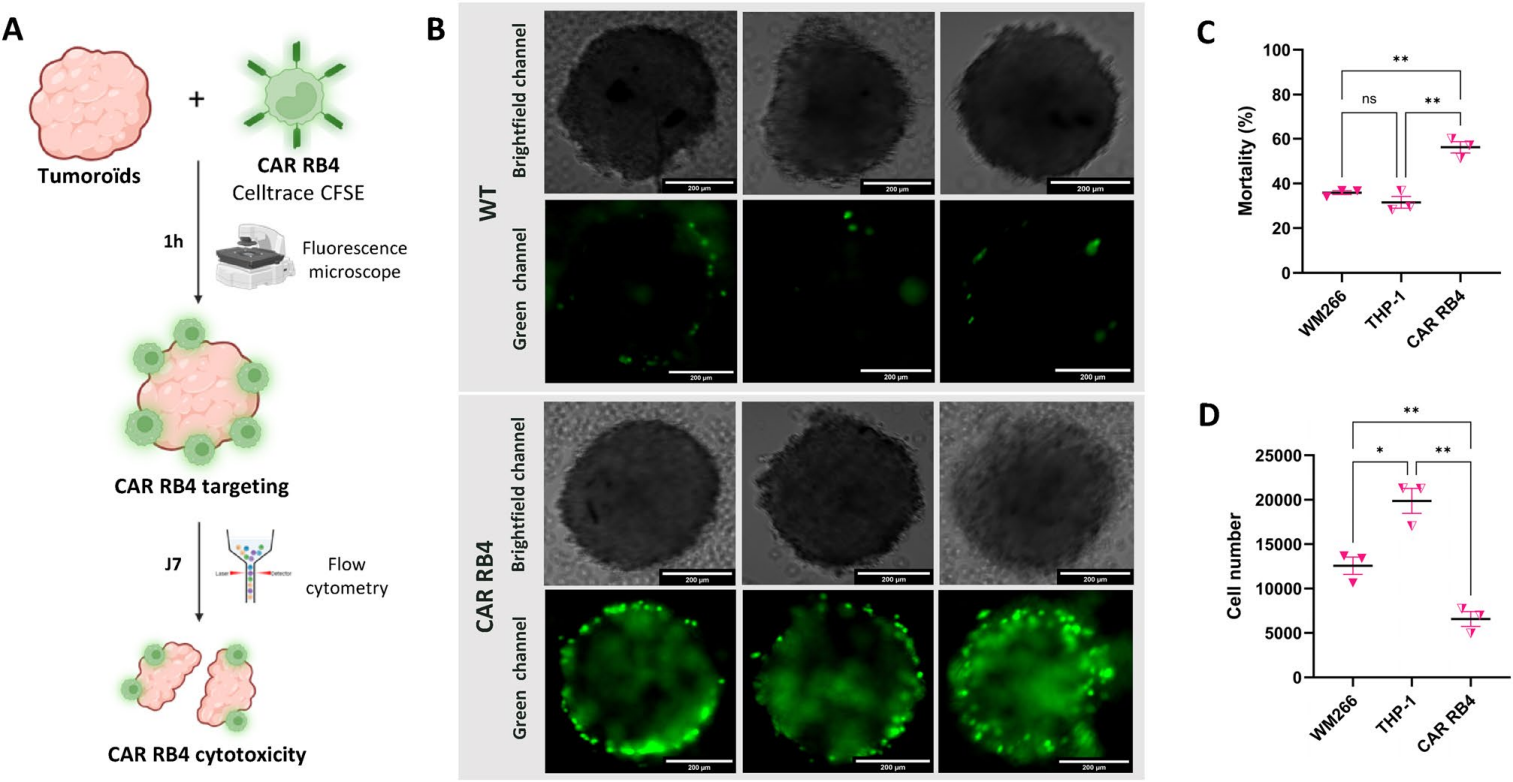
Evaluation of CAR-M RB4 targeting and cytotoxicity in a WM266 3D tumor model. **A** Schematic representations of the experimental approach to analyze phagocytosis and tumor growth in 3D model. **B** Fluorescence microscopy analysis of WM266 (ET_B_^High^) targeting by THP-1 cells with or without CAR RB4 in a 3D tumoroid model. Macrophages were pre-labeled with CFSE (green) and used at an E/T ratio of 5:1. Images were captured in green channel (488 nm) bright-field channel after 1 h of co-culture. **C** Flow cytometric analysis of tumoroid cell mortality using the viability marker 7-AAD. Differences were observed between CAR RB4 with all other conditions (WM266 vs. THP-1, *p*.value = 0.2375; WM266 vs. CAR RB4, *p*.value = 0.0092; THP-1 vs. CAR RB4, *p*.value = 0.0026). **D** Flow cytometry analysis of tumoroid cell growth. Differences were observed between all conditions (WM266 vs. THP-1, *p*.value = 0.0167; WM266 vs. CAR RB4, *p*.value = 0.0099; THP-1 vs. CAR RB4, *p*.value = 0.0029)

To evaluate the cytotoxicity in a more realistic model, tumor spheroids (tumoroids) were generated from the WM266 cell line. As shown in Fig. 5A, the targeting and cytotoxicity (tumor mortality and growth) of tumoroids were studied using CFSE-stained THP-1 or CAR RB4 macrophages.

Representative images acquired after 1 h of co-culture illustrate macrophages (THP-1 or CAR RB4) targeting of tumoroids (Fig. 5B). In the THP-1 condition, very little fluorescent signal is visible on the spheroid surface, in contrast to the CAR RB4 condition, where the spheroid surface shows a high fluorescent signal. These photographs demonstrate the intense targeting of CAR RB4 cells in a 3D model compared to unmodified macrophages.

Figure 5C and D shows cytotoxicity results after 7 days of co-culture in terms of mortality and growth, respectively. For cell mortality within the tumoroid (Fig. 5C), no significant difference is found between the WM266 condition alone and that cultured with THP-1 macrophages (WM266 vs. THP-1, *p*.value = 0.2375), with a mortality rate of approximately 32–35%. However, the CAR RB4 condition exhibited a notably higher mortality rate, with approximately 59.1% of cells dying within the tumoroid. This was evident in both the WM266 (WM266 vs. CAR RB4, *p*.value = 0.0092) and THP-1 (THP-1 vs. CAR RB4, *p*.value = 0.0026) conditions. These data demonstrate the exclusive cytotoxicity of CAR RB4 macrophages toward the tumoroid. Regarding tumoroid growth (Fig. 5D), the addition of THP-1 macrophages was associated with a significant increase in the number of tumoroid-forming cells (*p*.value = 0.0167). Indeed, the average density of a WM266 tumoroid is 12,500 cells (12,574 ± 1,682 cells) versus 19,900 (19,872 ± 2,440 cells) after co-culture with THP-1 macrophages. In the CAR RB4 macrophages co-culture condition, there was a significant reduction in the number of cells making up the tumoroid, with an average cell count of 6,500 (6,569 ± 1,441 cells) (WM266 vs. CAR RB4, p.value = 0.0099; THP-1 vs. CAR RB4, *p*.value = 0.0029, WM266 vs. THP-1, *p*.value = 0.0167). The addition of CAR RB4 is responsible for a 50% reduction in tumor cell numbers compared with the condition alone, unlike the THP-1 macrophage condition, which increases the number of tumor cells in the tumoroid.

## Discussion

The endothelin axis, and more precisely the ET_B_ receptor, plays a crucial role in melanoma and represents a promising therapeutic target for treatment of metastatic melanoma. Research carried out on the inhibition of this receptor using a selective antagonist, BQ788, has demonstrated the key role played by the endothelin axis in the expansion and migration of melanoma cells [27, 28]. It has also been demonstrated that a high expression of the EDNRB gene, which encodes for the ET_B_ receptor, is a poor prognostic indicator associated with a higher risk of brain metastases [29, 30]. Unfortunately, clinical studies on Bosentan, a mixed endothelin receptor antagonist, have failed to demonstrate therapeutic efficacy in Phase II trials [31]. These results underline the need to develop new non-antagonist strategies to target the endothelin axis in melanoma. In this context, we have developed an antibody library by DNA immunization, to specifically target the ET_A_ and ET_B_ receptors. Rendomab B4 (RB4) antibody has shown great potential in melanoma cell lines, notably by inhibiting cell proliferation following receptor internalization [3]. Therefore, RB4 antibody was selected for the development of our CAR-Macrophages approach.

Firstly, the RB4 scFv fragment, a crucial component of the CAR construct, was produced to assess its properties in comparison with the RB4 antibody. In terms of binding, the fragment presented similar properties to the full antibody, with specific binding to CHO-ET_B_ cells, as well as to melanoma cell lines WM266 and UACC257. The expression of the EDNRB gene, analyzed by RT-qPCR, correlates with the intensity of binding (Bmax_UACC257_ < Bmax_WM266_). The lack of recognition in the A375 cell line is coherent with the receptor’s low expression level.

Regarding affinity, although it is reduced for the scFv compared with the full antibody, it remains sufficient for application in CAR-based cell therapy. The study of Duan et al. showed that the reduced affinity of trastuzumab-derived scFv (0.58 nM → 3.2 nM) did not affect cell recognition and potentially reduced off-target toxicity on cells expressing low levels of HER2 [32].

The functionality of the scFv fragment on the cell surface was also confirmed by the targeting experiments: Only the UACC257 and WM266 cell lines were recognized, confirming the observations obtained with the fragment alone. One of the major challenges with CAR cell therapies is related to off-target toxicity on healthy cells that share the same receptor. The targeting of the ETB receptor in melanoma with an ADC (DEDN6526A) has already been addressed by Roche-Genentech in a Phase I/II clinical trial (NCT01522664). The conclusion of this study allowed a safe dose with an acceptable safety profile to be determined, and evidence of antitumor activity in patients to be shown [33]. A recent article published by our team studied off-target binding to tissues using the RB49 mAb, which also targets ETB and shares the N-terminal epitope with RB4. No off-target binding was detected in human healthy tissues. No off-target binding was detected in healthy tissues (e.g., liver and skin with normal vessels), whereas strong labeling was observed in all lymph node biopsies (e.g., melanoma cells and tumor endothelial cells) by immunohistochemistry (IHC). Moreover, in the preclinical models where our ADC was delivered, no adverse effects were observed and highly efficient antitumor activity was evidenced [34]. Both the RB4 and the RB49 target an allosteric epitope located on the N-terminal extremity of ETB, which is only accessible when ETB has bound endothelins, as is the case in a tumoral context where the endothelin axis drives tumor progression. This allosteric epitope-targeting RB4 property could limit the off-target effects of CAR-M. Future studies could determine the biodistribution of RB4 by immuno-PET imaging in healthy subjects and patients with melanoma to consider the scFv RB4 as being appropriate for targeting the ETB receptor in cell-based therapies. Therefore, it is possible to consider the scFv RB4 as being appropriate for targeting the ET_B_ receptor in cell-based therapies.

Using a 2D co-culture model, the antitumor activity of CAR RB4 was then analyzed. The results obtained are fully consistent with the expression profile of the ET_B_ receptor on melanoma cell lines. The lack of effect on A375 cells confirms that the response observed is specific to RB4 scFv-mediated recognition of the ET_B_ receptor. The increase in phagocytosis of WM266 cells results from the presence of the CD3*ζ* domain in the CAR construct. Although this domain is not naturally expressed in macrophages, several studies, including that of Morrissey et al. have shown that it induces an efficiency comparable to FcR*γ* or MEGF10 domains [21]. The presence of ITAM motifs in CD3*ζ* activates the PI3K pathway, promoting phagocytosis of target cells [35]. Concerning the reduction of tumor growth, this could result either from direct phagocytosis of tumor cells, or from the secretion of antitumoral cytokines. The CD28 co-stimulatory domain commonly used in CAR-T includes two functional motifs (YMNMTPRRP and PYAP) which activate the PI3K/AKT pathway, the NF-κB factor and inactivate the GSK3*β* effector [36]. Several studies demonstrate that activation of the PI3K/AKT axis is involved in cell survival[37], while NF-κB plays a central role in M1 polarization and the production of pro-inflammatory cytokines such as TNF-*α*, IL-1*β* and IL-6 [38, 39]. Busca et al. have also shown that the PI3K/AKT pathway and NF-κB induce expression of the anti-apoptotic factor BCL-XL [40]. Although further experiments are required to confirm this, it suggests a dual activity of the CD28 domain: promotion of cell survival (via BCL-XL) and induction of an inflammatory response (via IL-6, TNF-*α*, IL-1*β*). In the 3D co-culture model, the high localization of CAR RB4 macrophages on the periphery of WM266 spheroids also confirms the targeting efficacy. The absence of ET_B_^low^ spheroids is due to the A375 cell line’s inability to form spheroids. Cell viability and tumor growth were consistent with the 2D model. However, in contrast to the 2D model, an increase in tumor growth was observed in co-cultures containing unmodified THP-1 macrophages, suggesting a pro-tumor effect of these macrophages, promoting proliferation and inhibiting apoptosis. Introducing CAR RB4 appears to neutralize this process, probably by activating NF-κB and secreting pro-inflammatory cytokines. To understand the molecular mechanism of CAR RB4 signaling, it would be relevant to develop a CAR RB4 without the CD3*ζ* and CD28 domains to determine whether tumor growth remains similar to that induced by THP-1 macrophages. Analysis of the cytokine profile of the 3D model would also provide a better characterization of the polarization and activity of CAR RB4.

Based on the results obtained, RB4 scFv has great potential for targeting melanoma cells expressing the ET_B_ receptor. Into a THP-1 macrophage model, CAR RB4 demonstrated significant antitumor activity, with growth inhibition of over 50% in 2D and 3D co-culture models. These results support its potential use in treating cancers with high levels of ET_B_ expression, such as melanoma, ovarian cancer, pancreatic cancer and triple-negative breast cancer, as determined in a recent clinical trial using the ENB-003 antagonist and the role of ET_B_ in glioblastoma [41, 42]. However, the inherent limitations of the THP-1 model require validation of these results in primary macrophage. This step will be essential to assess CAR RB4 resistance in an immunosuppressive context closer to the pathophysiological conditions of solid tumors. In parallel, the efficiency demonstrated by the RB4 scFv suggests that it could also be integrated into a CAR-T model. Lui et al. have demonstrated a synergistic effect between CAR-T and CAR-M, notably via the production of pro-inflammatory cytokines and the activation of co-stimulatory pathways that reinforce cytotoxicity and persistence of CAR-T in solid tumors [43]. We have thus initiated a research program to evaluate a combined CAR-M/CAR-T approach using CAR RB4, to optimize the treatment of ET_B_⁺ solid tumors.

## Supplementary Information

Below is the link to the electronic supplementary material.

Supplementary file1 (PPTX 4866 KB)

## Data Availability

The datasets generated during and/or analysed during the current study are available from the corresponding author on reasonable request.
